# The “whole ingredients extract” of Astragali Radix improves the symptoms of dextran sulfate sodium-induced ulcerative colitis in mice through systemic immunomodulation

**DOI:** 10.1186/s13020-022-00661-0

**Published:** 2022-09-15

**Authors:** Ke-Gang Linghu, Qiushuo Ma, Shi-Hang Xiong, Mingming Zhao, Qiling Chen, Wen Xu, Meiwan Chen, Jian-Ye Zhang, Yuanjia Hu, Wei Xu, Hua Yu

**Affiliations:** 1 State Key Laboratory of Quality Research in Chinese Medicine, Institute of Chinese Medical Sciences, University of Macau, Macao SAR, China; 2 Macao Centre for Research and Development in Chinese Medicine, Institute of Chinese Medical Sciences, University of Macau, Macao SAR, China; 3 College of Pharmacy, Fujian University of Traditional Chinese Medicine, Fuzhou, Fujian China; 4 Key Laboratory of Molecular Target & Clinical Pharmacology and the State & NMPA Key Laboratory of Respiratory Disease, School of Pharmaceutical Sciences and the Fifth Affiliated Hospital, Guangzhou Medical University, Guangzhou, People’s Republic of China

**Keywords:** Astragali Radix, Whole ingredients extract, Immunomodulation, Ulcerative colitis

## Abstract

**Background:**

Ulcerative colitis (UC) is a common inflammatory intestinal disease. Astragali Radix (AR) is one of the traditional Chinese medicines used in clinic for UC treatment. In our previous study, the whole ingredient extract (WIE) from AR have been proved to possess better immunomodulatory effects on immunosuppressed mice compared with the conventional water extraction (WAE). In the present study, we further evaluated the therapeutic effects of WIE against dextran sodium sulfate (DSS)-induced UC in mice through systemic immune regulation.

**Methods:**

Gradient solvent extraction has been used to prepare the WIE of AR. The HPLC–MS analysis approach has been employed to analyze and compare the chemical differences between WAE and WIE. UC model was reproduced in 3% DSS-induced C57BL/6 mice for 6 days. Flow cytometric analysis for splenic lymphocyte subset. ELISA kits were used to determine the cytokines in the serum and colon tissues. The histopathological characteristics of colon were evaluated by hematoxylin–eosin staining and immunohistochemistry.

**Results:**

The chemical compositions and the contents of main active ingredients were more abundant and higher in WIE than those in WAE. The WIE treatment altered a better action on reducing colitis disease activity index (DAI) and histological scores, as well as the recovered body weight and increased colon length in mice compared to the WAE group. Additionally, WIE showed better effects in recovering the levels of peripheral white blood cells in blood and cytokines (IL-2, IL-6 and MCP-1) in serum or colon tissues, improving the percentage of CD3^+^ and the ratio of CD4^+^/CD8^+^ in the spleen, and inhibiting the spleen enlargement in DSS-induced UC mice.

**Conclusions:**

WIE has a more complete chemical composition than WAE. Meanwhile, WIE possesses better therapeutic effects on UC through resuming dysfunctional immunity in mice.

## Background

Ulcerative colitis (UC) is a chronic inflammatory bowel disease which happened in digestive system especially in the colon and rectum. Clinical symptoms are abdominal pain, diarrhea, bloody stool, etc. The progression of UC is a long-term process that is easily to relapse. The UC patients often undertake the high risk of colon cancer than ordinary people [1]. In the past decades, UC mainly happened in western countries, while the morbidity of UC is growing in Asian countries currently. The prevalence of UC has been becoming a noticeable problem in China [2]. Most scholars believe that UC is caused by multiple factors, including infection, immunity, genetic and mentality. Although the exact pathological mechanisms of UC are still not well understood, immune system dysfunction and severe inflammatory response at colon and rectum are widely observed for most UC patients [3].

Currently, the main clinical drugs used for treatment of UC are amino salicylic acid, glucocorticoid and immunosuppressant [4]. But these medicines could not cure UC completely, and the side effects are too severe. Extensive ongoing research efforts have been invested in developing the plant-based bioactive agents from traditional Chinese medicine for treating ulcerative colitis and made some achievements [5]. Conventionally, the water decoctions (water extracts, WAE) are used as main preparation for most Chinese medicines and health products. As a traditional tonic herbal medicine, Radix astragali (RA) has been used to strengthen human immunity in China for more than two-thousand years [6]. It is also one of the most frequently used herbs in treating UC [7] and is presented as the principal drug in various ancient prescriptions due to its functions in immunomodulation, such as the “Huangqi-huanglian pills” (*Puji Fang* in the Ming Dynasty by Zhu Su et al. 1361–1425 AD) and “Baizhu-Huangqi decoction” [8]. However, WAE mainly contains water-soluble components (polar macromolecules and small molecules), while alcohol-soluble components (weakly polar and non-polar small molecules) in the WAE are very limited. As a result, WAE contains incomplete chemical components compared to the herb itself, causing a loss of integrity in the corresponding pharmacological activity. Recently, astragalus polysaccharide (APS), as the main water-soluble component from RA, has attracted a lot of attention on UC treatment. Zhao et al. reported that APS attenuated rat experimental colitis by inducing regulatory T cells in intestinal Peyer’s patches [8]. Tian et al. revealed that APS alleviated murine colitis through inhibition of the NLRP3 inflammasome [9]. Lv et al. reported that APS protected against dextran sulfate sodium (DSS)-induced colitis by inhibiting NF-κB activation [10]. However, according to the current reports, main components of AR include astragalosides I-VII (saponins), polysaccharides, amino acids, flavonoids and trace elements, whether the APS could represent the whole function of AR on immune regulation and UC has not been investigated.

Previously, a “whole ingredients extract” (WIE) containing both polar and non-polar molecules of AR has been developed by our lab, and demonstrated to present better immunomodulatory effects than the WAE on cyclophosphamide-induced immunosuppressive mice [11]. In this study, the therapeutic effects of WIE and WAE against UC through immunomodulation were further investigated and compared on DSS-induced mice model.

## Methods

### Chemicals and reagents

Astragalosides (I, II, III and IV), calycosin-7-O-β-d-glucoside, ononin, calycosin and formononetin (the purities of all standards were higher than 98% by HPLC analysis) were purchased from Chengdu Pufeide Biotech Co., Ltd. (Chengdu, China). Acetonitrile (ACN) as HPLC grade was purchased from Merck (Darmstadt, Germany). All chemicals used were analytical grade and purchased from Sigma-Aldrich (St. Louis, MO, USA). Milli-Q water prepared from Sigma-Aldrich Co. (Millipore, MA, USA).

Anti-mouse CD3-FITC, anti-mouse CD4-APC, and anti-mouse CD8a-PE were provided by eBioscience (San Diego, CA, USA). Enzyme-linked immunosorbent assay (ELISA) kits IL-2, IL-6 and MCP-1 were supplied by Neobioscience Technology Co., Ltd. (Shenzhen, China). Primary antibody against COX-2 and the secondary antibody were purchased from Cell Signaling Technology (Danvers, MA, United States), all antibodies were diluted with 1:1000. Astragaloside I, Astragaloside II, Astragaloside III, Astragaloside IV (the purities of all standards were higher than 98% by HPLC analysis) were purchased from Chengdu Pefeide Biotech Co., Ltd. (Chengdu, China). Acetonitrile (ACN) purchased from Merck (Darmstadt Germany) is HPLC grade. And other chemicals used were analytical grade and purchased from Sigma-Aldrich (St. Louis, MO, USA).

### Herbs and herbal extracts

Astragali Radix (Astragalus, *Astragalus membranaceus* (Fisch.) Bge. var. mongholicus (Bge.) Hsiao) was purchased from Guangjitang CSPC Pharm Group (Guizhou, China). The herb sample was authenticated by the Dr. YU Hua (Macao, China) and the voucher specimen (HQ-2017001) was deposited in Institute of Chinese Medical Sciences, University of Macau.

The WIE and WAE were prepared with the methods as reported previously. For WIE, 400 g of powered AR was gradient-extracted with tenfold volume of 95% ethanol, 50% ethanol and water at 60 °C for 1 h for each, the filtered extracts were concentrated under reduced pressure, and then lyophilized using a Virtis freeze dryer. On the other hand, the WAE was prepared with the same protocol but using water instead of ethanol.

### HPLC–MS analysis

The contents of total polysaccharides in WIE and WAE were determined using phenol–sulfuric acid method [11].

The contents of Saponins (Astragaloside I~ IV) and flavonoids (calycosin-7-O-β-d-glucoside, ononin, calycosin and formononetin) were determined by a Waters Alliance HPLC system coupled with a Water ACQUITY QDa Mass Detector (Waters Corp., Milford, USA). Samples were eluted on a Waters Atlantis T3 column (150 mm × 2.1 mm, 3.0 µm) maintained at 25 °C. Elution was performed with a mobile phase of A (0.1% formic acid in water) and B (0.1% formic acid in ACN) under a gradient program: 0–2 min, 21% B; 2–12 min, 22–25% B; 12–20 min, 25–50% B; 20–30 min, 50–70% B. The flow rate was 0.4 mL/min, and the injection volume was 5 μL. The analytes were monitored by mass spectrometry with an electrospray ion source operating in the positive ion mode (ESI +) using single ion recording (SIR). The monitored ions were m/z 269.09 ([M + H]^+^, formononetin), 285.05 ([M + H]^+^, calycosin), 431.21 ([M + H]^+^, ononin), 447.13 ([M + H]^+^, calycosin-7-O-β-d-glucoside), 891.39 ([M + Na]^+^, astragaloside I), 849.44 ([M + Na]^+^, astragaloside II) and 807.30 ([M + Na]^+^, astragaloside III and IV), respectively. Between two injections, the column was washed with 100% B for 3 min and equilibrated with the initial mobile phase for 5 min.

### Experimental animals and treatments

Male C57BL/6 mice (22–24 g) supplied by the Faculty of Healthy Science Animal Centre of University of Macau were fed on a standard laboratory diet with free access to water at a controlled temperature of 22 ± 1 °C and relative humidity of 50% with a 12 h light/dark cycle. After one week of acclimatization, mice were randomly divided into six groups, control (CTRL), model (DSS, 3%), high dose of WIE (WIE-H, 3 g/kg), medium dose of WIE (WIE-M, 1.5 g/kg), low dose of WIE (WIE-L, 0.3 g/kg) and WAE group (WAE-H, 3 g/kg). Each group has 6 mice by random allocation. The control group was free to intake water for 14 days. Other groups were free to intake 3% dextran sulfate sodium (DSS) solution for 7 days, then changed to water for another 7 days. Extracts were administrated orally to the mice for 14 days in WIE and WAE groups. The body weight of each mouse was measured and recorded every day. All the experimental protocols were in accordance with the National Institutes of Health guidelines for the Care of Use of Laboratory Animals, and approved (reference No: UMARE-004–2020) by the Animal Research Ethics Committee, University of Macau, Macao SAR, China.

### Colon index and spleen index

In the end of experiment, mice were sacrificed by CO_2_ inhalation, the colon was dissected, and the length of the colon was measured using a ruler. Colon index (cm/g) = length of colon (cm)/body weight (g). The spleen was immediately excised and weighed. Spleen index (mg/g) = weight of spleen (mg)/body weight (g).

### White blood cell counting

Mice tail was wiped with warm water and disinfected with 75% alcohol. 5 μL of blood was collected from the tail vein and mixed with 95 μL of 0.2% acetic acid. Then the white blood cells were recorded with cell counting plate.

### ELISA assay for serum and colon

At the end of the experiment, blood samples were collected from the orbits of mice and centrifuged at 4,000 rpm for 10 min at 4 °C to obtain the serum sample. Additionally, the colon tissues in different groups were lysed by cell lysis buffer (Beyotime, Jiangsu, China), and the protein concentration was detected using a BCA protein assay kit (Thermo Fisher Scientific, MA, USA). Cytokines (IL-2, MCP-1, and IL-6) in the serum and lysate were measured using mouse ELISA kits according to the manufacturer’s protocol.

### Flow cytometry analysis

At the end of the experiment, spleens were isolated from mice and gently grinded and filtrated through a 40 μm strainer. Red blood cells were removed by red cell lysis buffer. Subsequently, 5–10 × 10^5^ splenocytes was incubated with FITC-CD3, APC-CD4 and PE-CD8a for 30 min at 4 °C in darkness and washed with PBS twice. The numbers of CD3^+^, CD4^+^ and CD8^+^ T lymphocytes was measured by LSRFortessa™ Flow Cytometer (BD, USA) and signified as percentage of total number of lymphocytes.

### Histopathology and immunohistochemistry

The colon tissue was fixed with 10% neutral phosphate buffered formalin for 7 days. After washing with tap water, the tissues were sequentially placed in gradient ethanol by standard methods [12], and processed in xylene-ethanol mixture (45 min)-xylene (20–60 min)-melted paraffin at 70 °C (2 h). The embedded colon tissue were cut into 6 μm sections on Paraffin Microtome (Thermo, UK).

Slices were deparaffinized and rehydrated with standard methods, then processed with Hematoxylin staining for 5–10 min, differentiated with 0.5% hydrochloric acid ethanol for 30 s, and stained with 0.5% Eosin for 10 s, following by rinsing with tap water for 25 min. Finally, the slices were dehydrated with standard protocol and sealed.

For immunohistochemistry assay, slices were deparaffinized and rehydrated by standard methods [12], and subjected to antigen retrieval by microwave (320 W, 11 min). The cyclooxygenase-2 was stained according to the commercial kit (Solarbio, Shanghai, China).

### Statistical analysis

All data from a minimum three experiments were presented as mean ± SD. Data were analyzed on GraphPad Prism 6.0 software based on a one-way ANOVA with Dunnet’s multiple comparisons test; *P* < 0.05 was considered difference significantly.

## Results

### Chemical characterization of herbal extracts

**Fig. 1:**
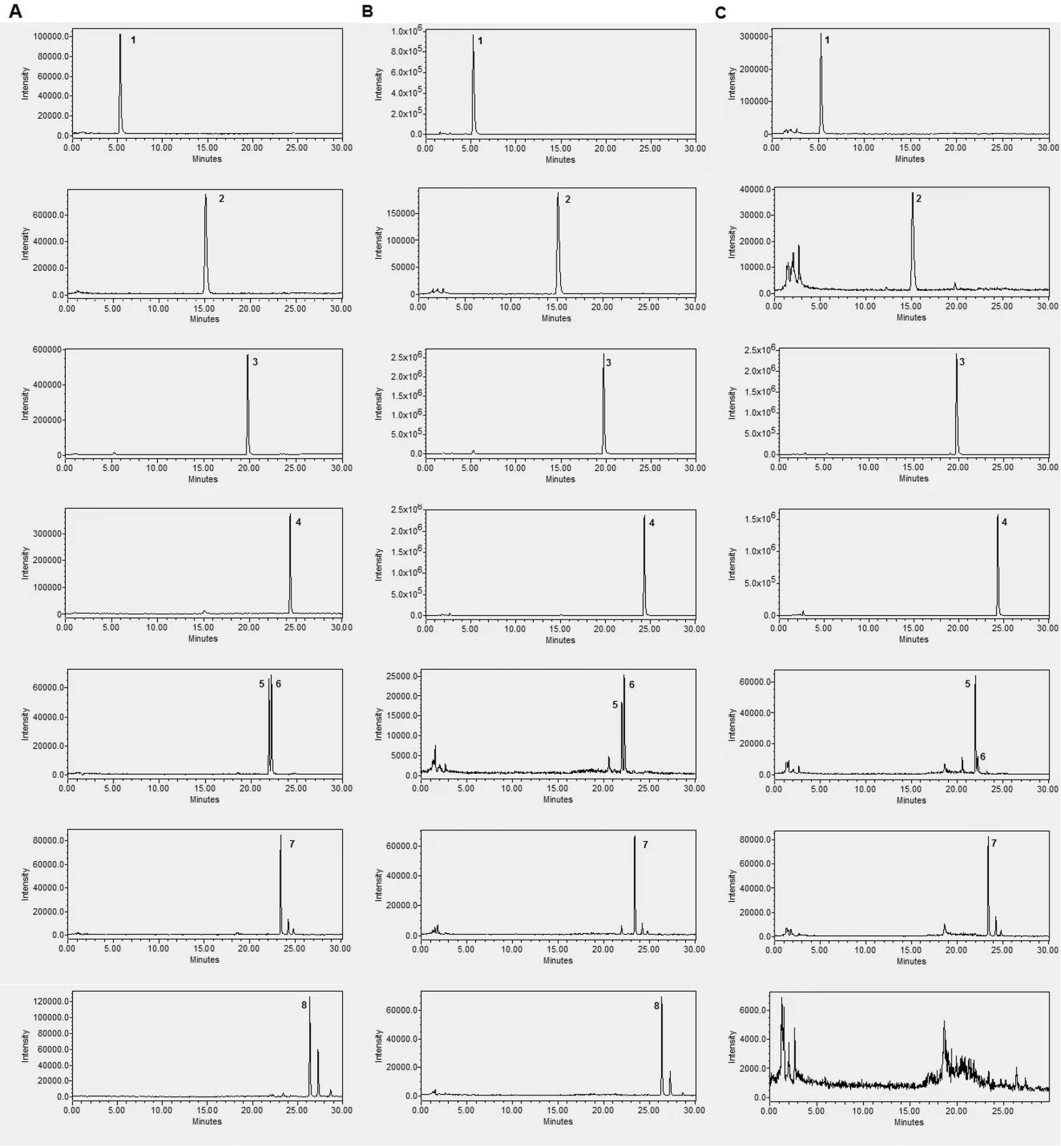
HPLC–MS chromatograms of **A** mixed standards, **B** WIE and **C** WAE. WIE: whole ingredient extract, WAE: water extract. 1: calycosin-7-O-β-d-glucoside, 2: ononin, 3: calycosin, 4: formononetin, 5: astragaloside III, 6: astragaloside III, 7: astragaloside II, 8: astragaloside I

The data for chemical characterization of WIE and WAE were summarized in Table 1. The results indicated that the similar extraction efficiencies of total extract and total polysaccharides for WIE (35.78% and 43.6%) and WAE (34.65% and 45.2%). However, the extraction efficiencies for the small molecules (flavonoids, Astragaloside I and Astragaloside III) of WIE were significantly higher to those of WAE, indicating the more abundance of small molecules in WIE (Fig. 1).

**Table 1:** Chemical characterization of WIE and WAE. data are presented as mean ± SD (*n* = 3)

Content (%, g/100 g extract)	WIE	WAE
Total polysaccharides	43.47 ± 0.35	44.63 ± 0.55
Calycosin-7-O-β-d-glucoside	0.0641 ± 0.0006	0.0191 ± 0.0002
Ononin	0.0151 ± 0.0001	0.0032 ± 0.0000
Calycosin	0.0311 ± 0.0001	0.0305 ± 0.0005
Formononetin	0.0478 ± 0.0007	0.0304 ± 0.0004
Astragaloside I	0.3676 ± 0.0063	N.D
Astragaloside II	0.0285 ± 0.0005	0.0360 ± 0.0004
Astragaloside III	0.0109 ± 0.0005	0.0047 ± 0.0002
Astragaloside IV	0.0091 ± 0.0001	0.0318 ± 0.0004

### The effects of WIE on food, liquid intake and animal growth

**Fig. 2:**
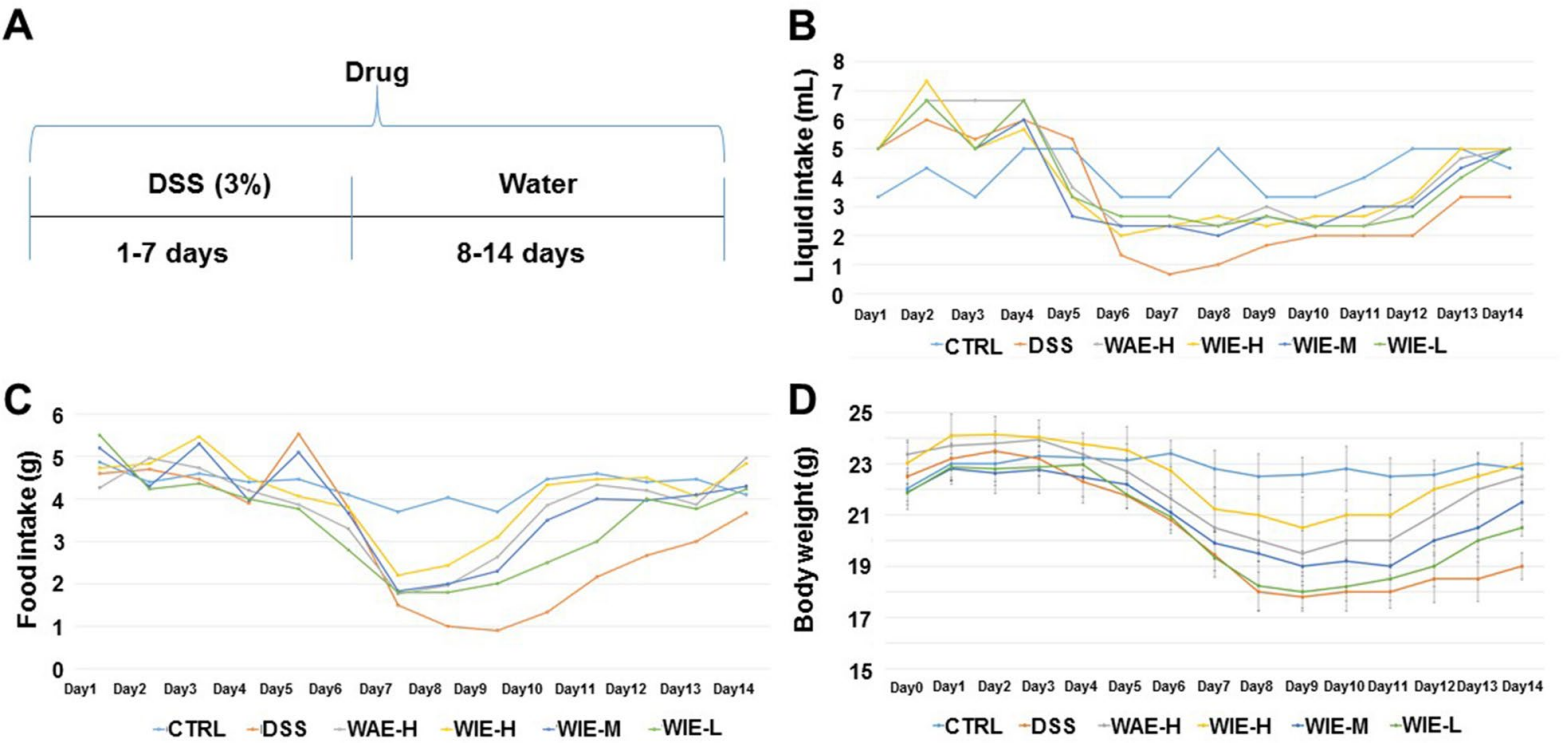
Food, liquid intake and body weight of experimental animals. **A** The route diagram of the whole experiment. **B** The changes of the liquid intake during the experiment period. **C** The changes of the food intake during the experimental period. **D** The changes of the body weight during the experiment period. Control (CTRL), model (DSS, 3%), high dosage of WIE (WIE-H, 3 g/kg), medium dosage of WIE (WIE-M, 1.5 g/kg), low dosage of WIE (WIE-L, 0.3 g/kg) and WAE group (WAE-H, 3 g/kg)

As shown in Fig. 2D, the mice in the control group keep the weight stable throughout the whole experiment. Compared with the control group, the body weight of the mice in the groups given DSS has been decreasing since the fourth day. The model group showed a dramatical decline till the ninth day (21% from the initial body weight), while the WAE and WIE treatment significantly reversed the decline of body weight of mice. WIE-H group showed the smallest body weight loss, followed by the WAE-H and WIE-M groups.

As shown in Fig. 2B, the liquid intake in the groups given DSS was higher than that in the water group in the first four days of the experiment. From the fifth day, the liquid intake in the DSS-treated groups began to decrease significantly, lower than that in water group. Eighth day’s treatment of AR extracts, the liquid intake of the treatment group was increased.

As shown in Fig. 2C, there was no obvious difference in food intake in the first five days of the experiment, while from the sixth day, the food intake in the DSS-treated groups were significantly reduced. The model group showed a dramatical decline till the ninth day. The AR extracts treatment group showed a short-term decline and began to recover from the seventh day. There is no obvious change of food intake in the control group during the whole experiment.

### WIE improved the disease activity index and colon index

**Fig. 3:**
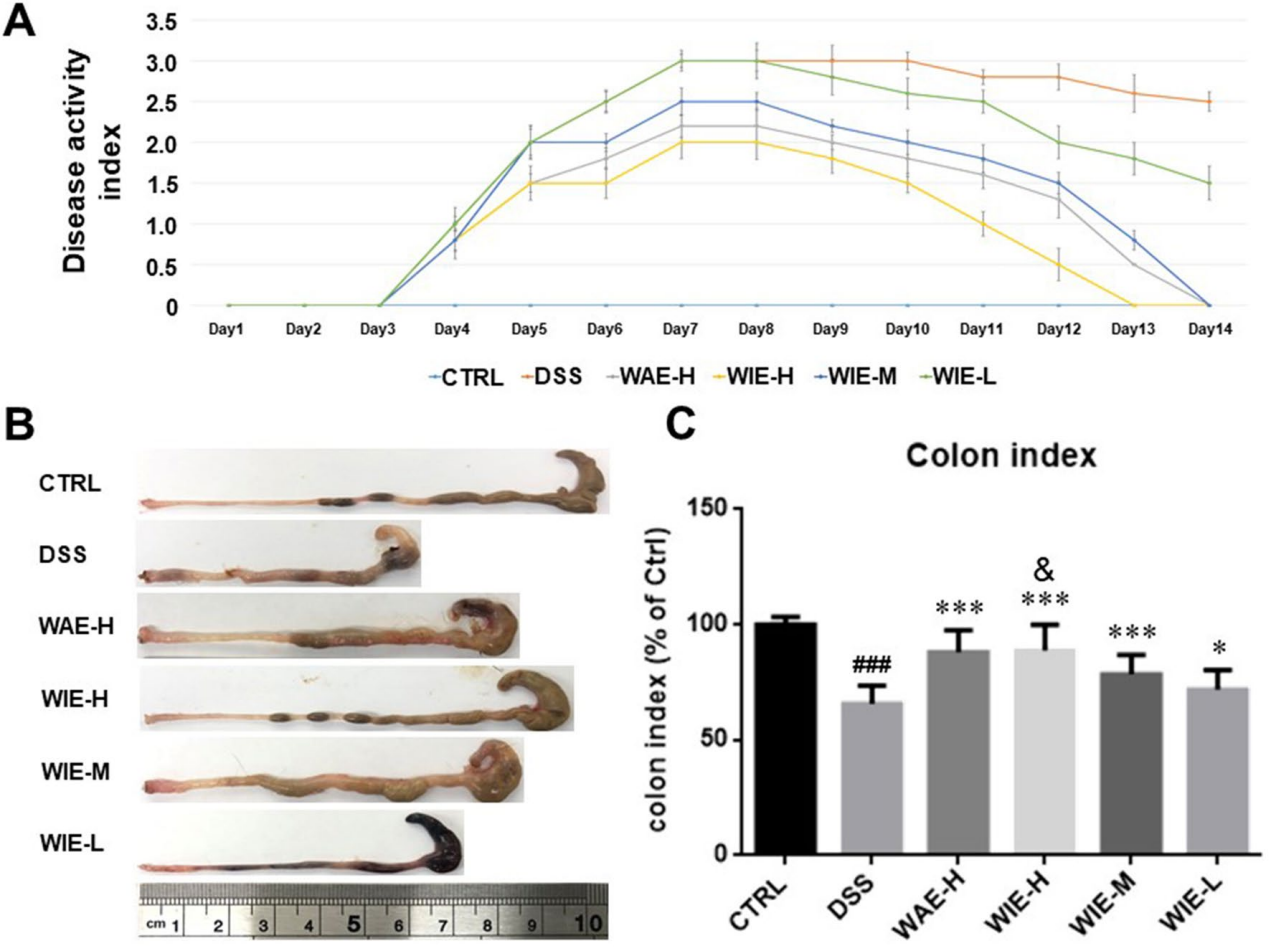
Effects of AR extracts on disease activity index (DAI) and colon index in DSS-induced UC. **A** The changes of the DAI during the experimental period. **B** Mice were sacrificed at day 15, the colon was dissected, measured, and imaged. **C** Colon index of different groups mice (Colon length/Body weight). Data are presented as Mean ± SD (*n* = 6). Control (CTRL), model (DSS, 3%), high dosage of WIE (WIE-H, 3 g/kg), medium dosage of WIE (WIE-M, 1.5 g/kg), low dosage of WIE (WIE-L, 0.3 g/kg) and WAE group (WAE-H, 3 g/kg). ^###^ *P* < 0.001 vs CTRL; ^***^ *P* < 0.001, ^*^ *P* < 0.05 vs DSS; ^&^ *P* < 0.05 vs WAE-H

A significant reduction in body weight and increased stool frequency were observed in DDS-treated mice and the stools were loose or watery containing some mucus and/or blood. DSS group mice with colitis display significant increase of DAI (Fig. 3A), while treatment with WIE in high dose significantly reversed the DAI, followed by WAE-H, WIE-M and WIE-L group.

To investigate the therapeutic effects of AR extracts on UC, the colon lengths in different groups were recorded and compared. A representative colonic appearance of mice in each group was shown in Fig. 3B. The mouse colon in DSS group was significantly shortened compared with the control group. AR extracts treatment alleviated DSS-induced colon shortening (Fig. 3C) and WIE-H showed a better effect than WAE-H and WIE-M. While the low dose of WIE did not improve the length of colon obviously, compared with the DSS group.

### WIE reduced the production of IL-6 and MCP-1 while increased the IL-2 in serum and colon of DSS-induced mouse

**Fig.4:**
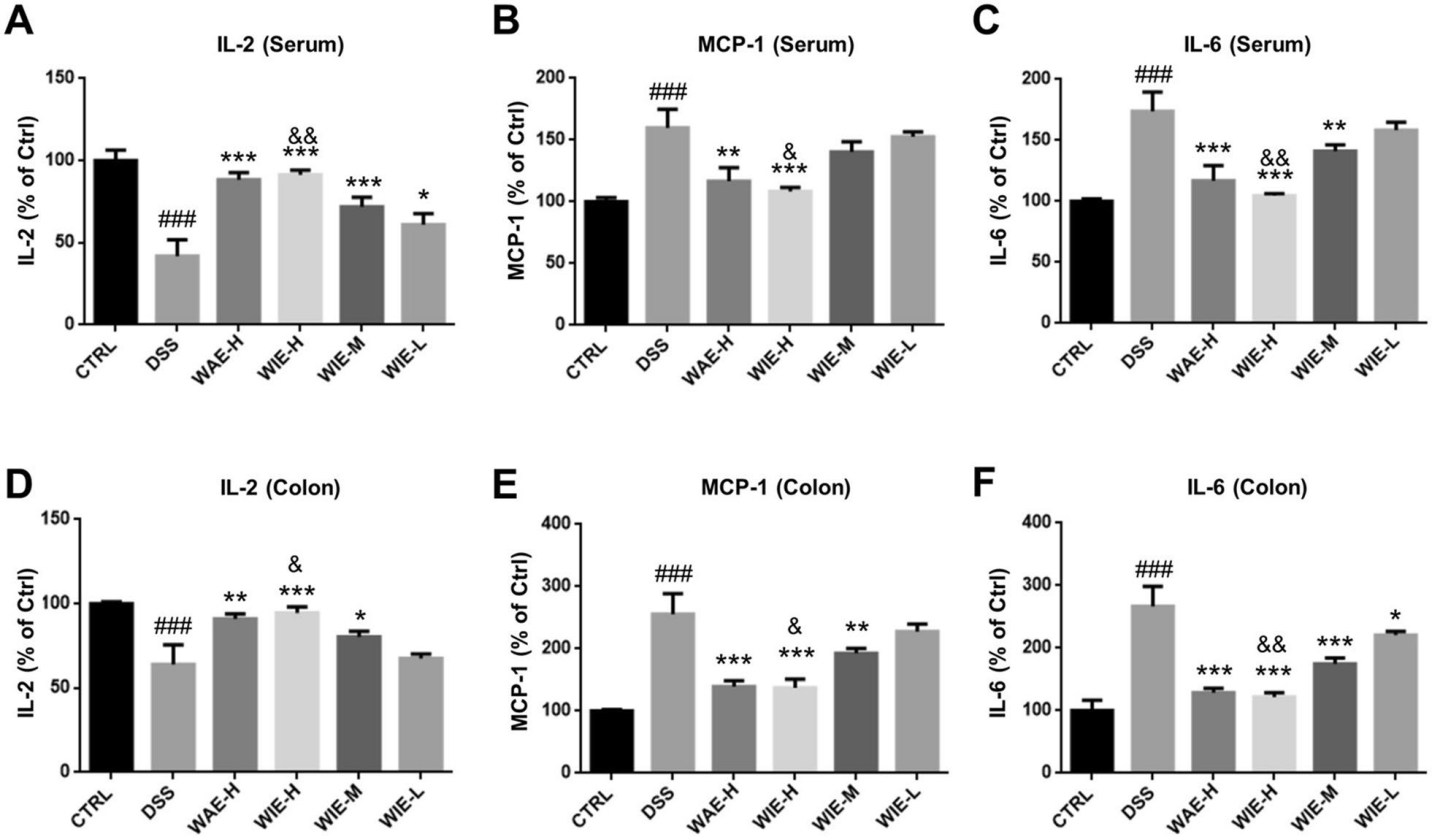
Effects of AR extracts on cytokines in serum and colon in DSS-induced mice. IL-2 levels in serum (**A**) and colon (**D**) of DSS-induced UC mice. MCP-1 levels in serum (**B**) and colon (**E**) of DSS-induced UC mice. IL-6 levels in serum (**C**) and colon (**F**) of DSS-induced UC mice. Data are presented as Mean ± SD (*n* = 6). Control (CTRL), model (DSS, 3%), high dosage of WIE (WIE-H, 3 g/kg), medium dosage of WIE (WIE-M, 1.5 g/kg), low dosage of WIE (WIE-L, 0.3 g/kg) and WAE group (WAE-H, 3 g/kg). ^###^ *P* < 0.001 vs Ctrl; ^***^ *P* < 0.001, ^**^ *P* < 0.01, ^*^ *P* < 0.05 vs DSS; ^&&^ *P* < 0.01, ^&^ *P* < 0.05 vs WAE-H

As shown in Fig. 4, the expressions of MCP-1 and IL-6 were markedly increased in DSS-induced mice, while treatment with AR extracts significantly reduced MCP-1 and IL-6 expression in colon tissues and serum, especially in the WIE-H, WAE-H, and WIE-M groups. IL-2 level was reduced significantly in DSS-induced mice, which was increased after treatment with AR extracts both in tissues and serum.

### WIE increased white blood cell number while decreased spleen index

**Fig.5:**
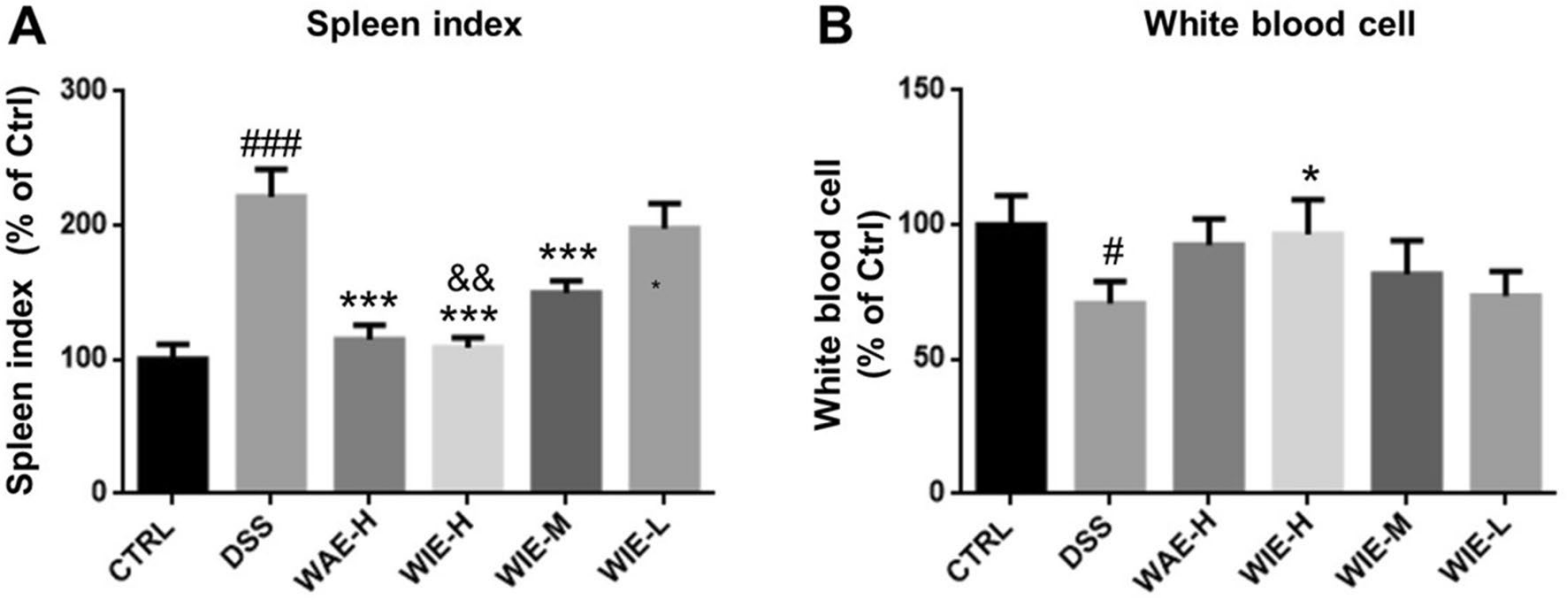
Effects of AR extracts on spleen index and peripheral blood white cells in DSS-induced mice. **A** Effects of AR extracts on spleen index of DSS-induced UC mice. **B** The counts of white blood cells in DSS-induced UC mice. Data are presented as Mean ± SD (*n* = 6). Control (CTRL), model (DSS, 3%), high dosage of WIE (WIE-H, 3 g/kg), medium dosage of WIE (WIE-M, 1.5 g/kg), low dosage of WIE (WIE-L, 0.3 g/kg) and WAE group (WAE-H, 3 g/kg). ^###^ *P* < 0.001, ^#^ *P* < 0.05 vs CTRL; ^***^ *P* < 0.001, ^*^ *P* < 0.05 vs DSS; ^&&^ *P* < 0.01 vs WAE-H

White blood cell and spleen index represent the systemic immune function in a mouse. As shown in Fig. 5A, the spleen index of mice in DSS group was significantly higher than that of the control group, which means the enlargement of spleen after inducement with DSS in mice. However, after treatment with AR extracts, the spleen index in the WIE-H, WAE-H and WIE-M groups were decreased significantly. As shown in Fig. 5B, the number of peripheral white blood cell (PWBC) in DSS-induced mice were markedly reduced. After treatment with AR extracts, there is an obvious recovery of PWBC numbers, especially the WIE-H could significantly enhance the PWBC numbers in UC mice.

### ***WIE upregulated the level of CD3***^+^***and CD4***^+^***/CD8***^+^***T lymphocytes in spleen***

**Fig.6:**
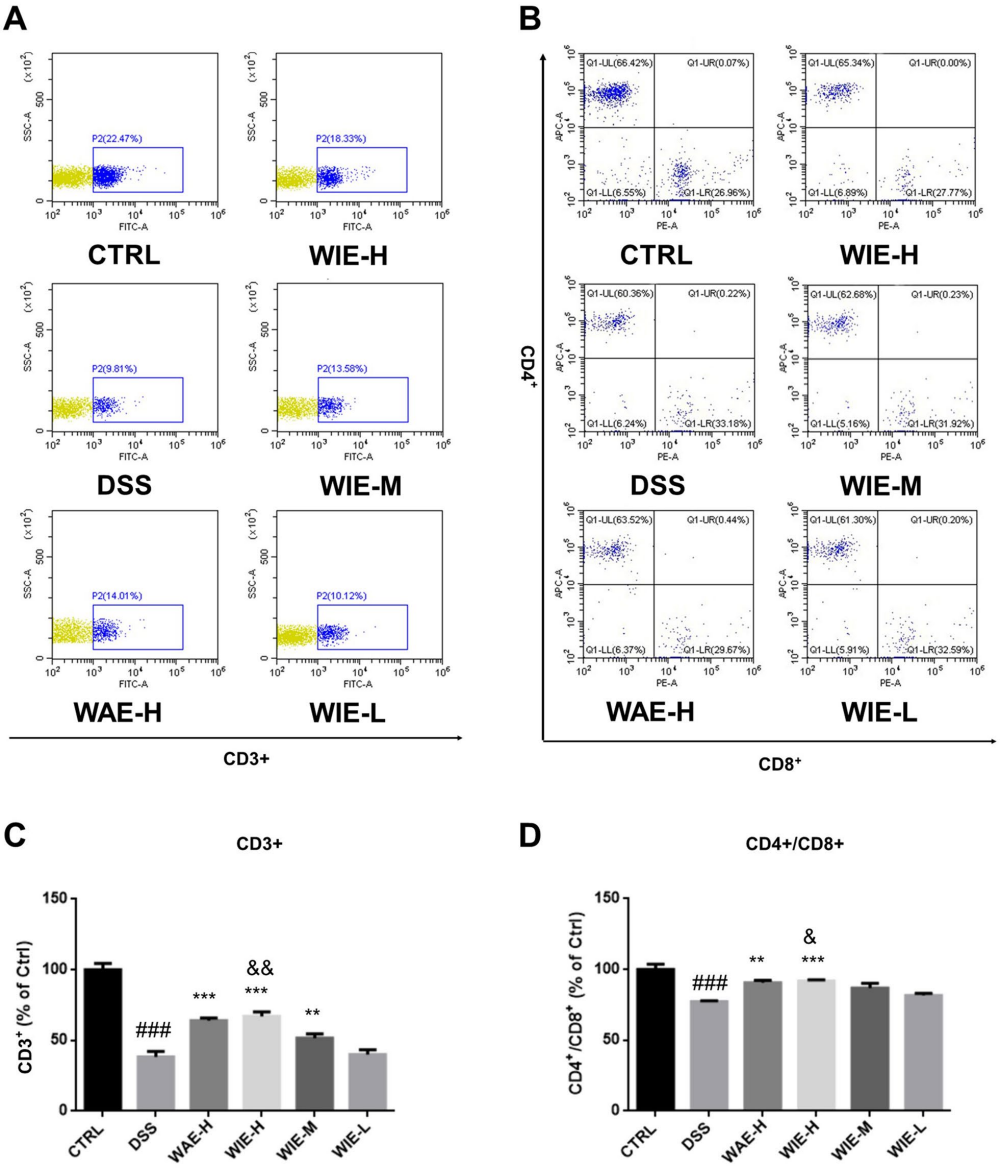
Counts of T lymphocytes determined by flow cytometry. **A** Representative images of CD3^+^ counting determined by flow cytometry for mice treated by water, DSS, WAE and WIE. **B** Representative images of CD4^+^ and CD8^+^ counting determined by flow cytometry for mice given by water, DSS, WAE and WIE. Proportion of CD3^+^ (**C**) and ratio of CD4^+^/CD8^+^ (**D**) in each group. Data are presented as Mean ± SD (*n* = 5). Control (CTRL), model (DSS, 3%), high dosage of WIE (WIE-H, 3 g/kg), medium dosage of WIE (WIE-M, 1.5 g/kg), low dosage of WIE (WIE-L, 0.3 g/kg) and WAE group (WAE-H, 3 g/kg). ^###^ *P* < 0.001, ^#^ *P* < 0.06 vs Ctrl; ^***^ *P* < 0.001, ^**^ *P* < 0.01 vs DSS; ^&&^ *P* < 0.01, ^&^ *P* < 0.05 vs WAE-H

As shown in Fig. 6, immunophenotype of splenocytes were evaluated by counting CD3^+^, CD4^+^ and CD8^+^ T lymphocytes using flow cytometry analysis. The percentages of CD3^+^ lymphocytes (Fig. 6A and C), and the ratio of CD4^+^/CD8^+^ were determined to be significantly decreased in DSS-treated mice (Fig. 6B and D). Treatment with WIE-H and WAE-H could increase the percentages of CD3^+^ and the CD4^+^/CD8^+^ ratio significantly.

### The effects of WIE on histopathological evaluation

**Fig.7:**
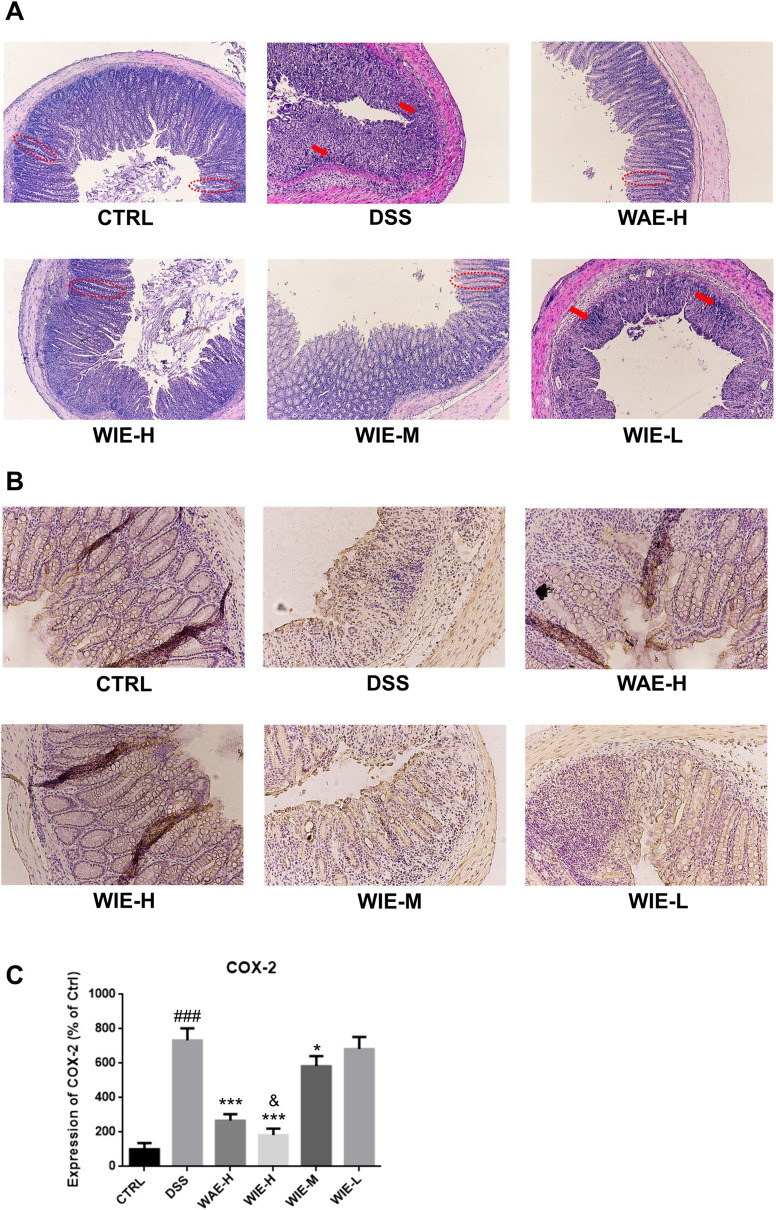
Effects of AR extracts on histopathological characterization of colon in DSS-induced mice. **A** Representative H&E staining images from the six different groups (× 100), red box indicates the intact crypt structure in colonic mucosa and red arrows indicated the inflammatory cells infiltration. **B** Representative COX-2 immunohistochemistry staining images from six different groups (× 100). **C** Relative quantification for the COX-2 expression (*n* = 5). Control (CTRL), model (DSS, 3%), high dosage of WIE (WIE-H, 3 g/kg), medium dosage of WIE (WIE-M, 1.5 g/kg), low dosage of WIE (WIE-L, 0.3 g/kg) and WAE group (WAE-H, 3 g/kg). ^###^ *P* < 0.001 vs CTRL; ^***^ *P* < 0.001, ^*^ *P* < 0.05 vs DSS; ^&^ *P* < 0.05 vs WAE-H

The histopathological results of colonic mucosa in each group of mice were shown in Fig. 7A. It could be seen from the control group that the colon mucosa tissue and crypt structure was intact, and no edema was observed in the submucosa. In the DSS group, the mucosal epithelium was severely damaged and shed, the crypt structure was disappeared, and a large number of inflammatory cells were infiltrated into submucosa. Treatment with the WIE-H, WIE-M and WAE could alleviate the above pathological condition.

COX-2 is a critical protein associated with the inflammation in colon mucosa tissue. To further evaluate the protective effect of Astragalus extracts on DSS-induced UC, we detected the expression of COX-2 in colon tissue with immunohistochemical analysis. As shown in Fig. 7B and C, a large number of COX-2 positive cells were observed in the colon mucosa of DSS-treated mice. After treatment with AR extracts, the COX-2 positive cells were significantly reduced, and WIE-H group showed the most significant inhibition effect, followed by the WIE-M and WAE-H.

## Discussion

Ulcerative colitis (UC) is a chronic inflammatory intestinal disease, immune system dysfunction and severe inflammatory response at colon and rectum are the typical characteristics. In recent years, several traditional Chinese medicines (TCM) have been shown to protect against UC [5]. Astragali Radix (AR) is a popular Chinese medicine, which has been traditionally used to promote the immune function [13]. “Baizhu-Huangqi decoction”, a traditional Chinese medicine formula consisted with AR and another Chinese medicine has been used for UC treatment. Based on the traditional use of water decoction (water extracts, WAE), many modern researchers focused their study on the water extract of herb medicine, astragalus polysaccharide (APS) is one of the main bioactive components from water extracts of AR and has been revealed to show anti-colitis effects [14]. Astragaloside IV (ASI) is a monomeric compound identified from APS and also been confirmed to exert anti-UC effects in vivo and in vitro [15]. However, because of the different physical and chemical properties, the alcohol-soluble components (weak polar and nonpolar small molecules) in water extract are very limited. As a result, compared with the herbal medicine itself, WAE contains incomplete chemical components, leading to a decrease in the integrity of the corresponding pharmacological activity. Therefore, our research group developed the whole ingredient extract (WIE) method, trying to retain the small molecule compounds in AR to make its ingredients more complete. Further study showed that WIE of AR is better immune recovery than WAE in cyclophosphamide-induced immunosuppressive mice. In this study, we investigated and compared the immune regulation effects between the WIE and WAE on DSS-induced UC model.

The spleen belongs to the peripheral immune organs and is the largest lymphoid organ in the human body, which plays important roles in regard to red blood cells (erythrocytes) and the immune system. Many studies have initially detected the state of the body's immune function by detecting changes in the spleen weight index. As shown in Fig. 5A, our results showed that the spleen index of the DSS group is much higher than that of the control group, it suggested that the dysfunction of the spleen in DSS group. Compared with the DSS group, the spleen index in WIE-H, WIE-M and WAE-H groups was declined, indicating that AR extracts could improve the spleen function to enhance the immunity of UC mice. Similarly, results from Fig. 5B indicated that, compared with the control group, the white blood cells (WBCs) in the DSS group are reduced, suggesting the suppressed immune function in the UC mice, treatment with AR extracts could increase the level of WBCs, and the WIE-H showed a better effect than WAE-H.

There are two types of acquired immune systems including cellular immunity and humoral immunity, and cellular immunity is dominant. T lymphocytes are the most important regulatory components in cellular immunity. T lymphocytes are not only effector cells of cellular immunity, but also play an important role in regulating the immune response. T cells are grouped into a series of subsets based on their function, CD3 is present on all mature T cell membranes, CD8 is distributed on the surface of killer T cells, and CD4 is distributed on the helper cells. The proportion of CD3^+^ T cell and the ratio of CD4^+^/CD8^+^ are critical parameters for immune system. As shown in Fig. 6, DSS treatment not only induced the decrease of proportion of CD3^+^ T cell but also a significant decline of the ratio of CD4^+^/CD8^+^, suggesting weak immune function in the UC mice. However, this immune-suppressed status was reversed after the treatment of the AR extract, and WIE-H showed a better modulatory effect than that of WAE-H (Fig. 5).

CD4^+^T cells secrete a variety of cytokines after activation. According to the different cytokines, T helper cells are divided into two functional subgroups, Th1 and Th2. Th1 mainly produces IL-2, IFN-γ, TNF etc. Th2 mainly produces IL-4, IL-5, IL-6, IL-10 etc. [16]. IL-2 is the most important and powerful T cell growth factor in the body, and it is a key factor to ensure the body's normal immune function [17]. IL-2 is produced by activated Th1 lymphocytes and stimulated macrophages, natural killer (NK) cells, and cytotoxic T cells in cell-mediated immune responses. Figure 4A and D showed that the IL-2 levels in serum and colon tissue of DSS mice were lower than those in the control group, but increased in WIE-H, WIE-M and WAE-H groups, indicating that AR extracts could attenuate the immune function through balancing the level of IL-2.

IL-6 is a common pro-inflammatory cytokine. It has been shown that serum levels of this pro-inflammatory cytokine were significantly increased in IBD patients [18]. Further research found a correlation between IL-6 expression and disease activity in patients with Crohn's disease (CD) and UC [19]. Louis proposed that high serum levels of IL-6 could be used as a prognostic marker for relapse in patients with resting CD [20]. Consistent with these reports, our results showed that, in the serum and colon tissue of DSS group, the expression of IL-6 was significantly higher than that of the control group, which could be decreased after the administration with WIE-H, WIE-M and WAE-H (Fig. 4C and F), indicating that IL-6 may participate in the regulatory process of AR on UC.

Studies have confirmed that the expression of MCP-1 in UC lesions is higher than that in normal mucosa. Increased expression of MCP-1 was also observed in the lesions of CD patients, and the expression of MCP-1 was positively correlated with the severity of UC. In addition, recent studies have shown that mice with MCP-1 receptor deficiency are given DSS to induce experimental colitis animal models, and the number of ulcers in the intestine and the degree of inflammation were reduced compared to normal mice. These illustrate the important role of MCP-1 in the pathogenesis of UC [21]. As shown in Fig. 4B and E, the expression of MCP-1 in the intestinal tissues and serum of the DSS group was significantly increased compared to the control group. The expression of MCP-1 in the WIE-H, WIE-M and WAE-H were lower than that in the DSS group, which demonstrated that AR extracts could reduce the inflammatory response in the UC model by down-regulating the MCP-1 expression.

Abovementioned content showed the immune-improvement effects of AR extracts, and the WIE exhibited a better effect than WAE. Then, we made an assessment on therapeutic effects of UC through the body weight, disease activity index, colon length, and histopathological analysis. Clinical studies have shown that UC patients will develop diarrhea and bloody Stool [22]. Consistent with clinical studies, the mice in the model group gradually began to lose weight and accompany blood in the stool after DSS treatment. AR extracts treatment could significantly attenuate the weight loss of mice (Fig. 2D) and reduce the blood in the stool (Fig. 3A). Continuous treatment of DSS lead to shortening of colon length accompanying with inflammation and edema of the colon [23]. However, after treatment with AR extract, colon shortening was significantly reduced (Fig. 3B). In UC model mice, colonic mucosal tissue often showed an obvious edema, hyperemia, shedding of epithelium, disappearance of crypt structure, and infiltration of inflammatory cells [24]. Consistent with these reports, in our result, the mice in the model group also exhibited the above pathological features. AR extracts could alleviate the above symptoms and reduce the pathological score of mice (Fig. 7A–C).

## Conclusions

In summary, WIE-AR is more complete than WAE-AR in chemical integrity, and the therapeutic effect of WIE-AR on UC by resuming the immune function are better than WAE-AR at the same dose in mice. This study suggests that WIE might be a better method than WAE to represent the whole function of some herb medicines.

## Data Availability

The datasets used and/or analyzed during the current study are available from the corresponding author on reasonable request.

## Abbreviations

APS: Astragalus polysaccharide
AR: Astragali Radix
DAI: Disease activity index
DSS: Dextran sulfate sodium
IL-2: Interleukin-2
IL-6: Interleukin-6
MCP-1: Monocyte chemoattractant protein-1
PBS: Phosphate buffered saline
PWBC: Peripheral white blood cell
UC: Ulcerative colitis
WAE: Water extract
WIE: Whole ingredients extract
